# A transformers-based approach for fine and coarse-grained classification and generation of MIDI songs and soundtracks

**DOI:** 10.7717/peerj-cs.1410

**Published:** 2023-06-19

**Authors:** Simone Angioni, Nathan Lincoln-DeCusatis, Andrea Ibba, Diego Reforgiato Recupero

**Affiliations:** 1 Department of Mathematics and Computer Science, University of Cagliari, Cagliari, Sardegna, Italy; 2 Department of Music, Fordham University, New York, United States of America

**Keywords:** Classification, Deep Learning, Generation, MIDI, Transformers

## Abstract

Music is an extremely subjective art form whose commodification *via* the recording industry in the 20th century has led to an increasingly subdivided set of genre labels that attempt to organize musical styles into definite categories. Music psychology has been studying the processes through which music is perceived, created, responded to, and incorporated into everyday life, and, modern artificial intelligence technology can be exploited in such a direction. Music classification and generation are emerging fields that gained much attention recently, especially with the latest discoveries within deep learning technologies. Self attention networks have in fact brought huge benefits for several tasks of classification and generation in different domains where data of different types were used (text, images, videos, sounds). In this article, we want to analyze the effectiveness of Transformers for both classification and generation tasks and study the performances of classification at different granularity and of generation using different human and automatic metrics. The input data consist of MIDI sounds that we have considered from different datasets: sounds from 397 Nintendo Entertainment System video games, classical pieces, and rock songs from different composers and bands. We have performed classification tasks within each dataset to identify the types or composers of each sample (fine-grained) and classification at a higher level. In the latter, we combined the three datasets together with the goal of identifying for each sample just NES, rock, or classical (coarse-grained) pieces. The proposed transformers-based approach outperformed competitors based on deep learning and machine learning approaches. Finally, the generation task has been carried out on each dataset and the resulting samples have been evaluated using human and automatic metrics (the local alignment).

## Introduction

Artificial intelligence (AI) is one of the current hot technologies within research communities, daily life, and business. It also represents an important driver for the creation and the future dissemination of the metaverse ecosystem Lee et al (2021). It affects several domains and is used wherever there is the need to provide an answer automatically. Music is one of such domains and one of the most known tools which leverages AI in the music domain (to identify songs from a small sample) is represented by Shazam (https://www.shazam.com/home) or the music discovery algorithms of Spotify (https://www.spotify.com/). The growing number of industrial applications of music information retrieval has provided the availability of large amounts of music data and rich diversity of genres with the related tasks of their automatic classification Qiu, Li & Sung (2021). Due to such availability of music data, deep learning approaches have taken over and have been able to achieve impressive performances on classification and data generation tasks. At a higher level, deep learning approaches outperformed state-of-the-art machine learning approaches in several tasks where data are represented as text Atzeni & Recupero (2020), images Chai et al. (2021), and time-series Carta et al. (2021); Barra et al. (2020) such as financial or audio data. Music is indeed a type of time-series data and can therefore benefit from the development of cutting-edge approaches within the AI sphere.

Identifying and classifying music by genre have typically been the domain of trained musicologists who can quickly identify timbral, rhythmic, harmonic, and melodic signifiers by ear and use these to label a given piece or excerpt. With the rise of AI technologies, this has changed as well. Today we already have the technology to automatically solve several tasks which have historically been performed by people. An AI music classifier or an AI composer were terms used only in old science-fiction movies or books but they are reality today Carnovalini & Rodà (2020).

For machines, classifying and generating music by genre has always been a challenging task especially if we consider the massive amounts of music data and the highly heterogeneous type it can be: pop music, rock, jazz, blues, classical, musical theatre, electronic, heavy metal, children’s instrumental, soundtracks, etc. Differences between music types can include many characteristics: for the traditional genres, style features might be more defined whereas for some sub-genres patterns might be less recognizable even for a musicologist.

Musical data can be encoded through a digital or analog recording, or more indirectly through a musical notation system. The first one records the audio intensity over time based on sound signals like an MP3 or an AIFF audio file for example. They contain directly all the sounds and are not easy to edit. In any case, recent works have shown how to learn powerful representations from this kind of audio (Baevski et al., 2020). The second one is a symbolic representation through a musical score. MIDI (MIDI Association, 2023) is a symbolic music format equivalent to a written musical score and gives specific information about pitch, duration, volume, and tempo, but contains no sound itself. Audio would represent the physical recording of the sound. It consists of a protocol that allows musical information to be shared between electronic musical devices and computers. When using MIDI format, the pitch, duration, start time, and velocity (loudness) of each note and chord are stored in a file. This is exactly analogous to a traditional notated musical score, except the information is stored in a way that is easily analyzed by computers as opposed to human musicians. Each of these features may be inspected to try to identify the music genre or to generate new musical patterns. One convenient characteristic of MIDI files is their size, usually very small. Moreover, MIDI files provide a higher level of detail on music elements, they are versatile and controllable and allow researchers and authors to better understand the music represented. Given their characteristics, MIDI files can be considered as a kind of string-type data in a time series format. This allows us to adopt natural language processing (NLP) techniques (Dridi, Atzeni & Recupero, 2019; Dridi & Recupero, 2019) to them. Several successful efforts have been already done for classification and generation tasks related to MIDI music (Cataltepe, Yusuf & Sonmez, 2007; Jedrzejewska, Zjawinski & Stasiak, 2018; Gunawan, Iman & Suhartono, 2020; Payne, 2019). Authors have always used a combination of feature extraction techniques and deep learning methods and come up with promising results. As previously mentioned, this was possible thanks to the high availability of labeled MIDI music data that allow machine and deep learning approaches to perform well. At the same time, transformers have seen a marked increase in use in the last couple of years. They were dis covered in Vaswani et al. (2017) and were able to beat several state-of-the-art approaches on different NLP tasks. They have been employed in machine language translation (Sefara et al., 2021), conversational agents (Golovanov et al., 2020), sentiment analysis (Pipalia, Bhadja & Shukla, 2020), language generation (Varshney et al., 2020), text summarization (Luo, Guo & Guo, 2019) and so on bringing a huge advancement to the literature in different tasks and domains. Transformers have been already successfully applied to perform music genre classification and generation (Qiu, Li & Sung, 2021; Huang et al., 2018).

In this article, we follow this direction and want to further analyze the effectiveness of transformers for both classification and generation tasks at fine and coarse-grained levels, and especially when there are not enough labeled data samples. One example occurs with MIDI soundtracks used in old videogames. We are interested to explore this issue and, as such, in this article, we consider the Nintendo Entertainment System soundtracks together with two more datasets of music. The Nintendo Entertainment System Music Database (NES-MDB) (https://github.com/chrisdonahue/nesmdb; Donahue, Mao & McAuley, 2018) is a dataset intended for building automatic music composition systems for the NES audio synthesizer. It contains 5,278 soundtracks of 397 NES games. The soundtracks consist of a combination of more than two million notes. We have manually annotated the soundtracks of a subset of it consisting of 133 games in five genres of video games: Role Playing Games (RPG), Sport, Fighting, Shooting, Puzzle. In video games, the music affects the gamer experience, and even if in a certain game there are different music genres, we are interested in inspecting how the different types of music can share similar patterns with respect to the video game genre that it is on. One more dataset consists of MIDI files of rock songs from the following artists: Eric Clapton, Queen, Beatles, and Rolling Stones. We downloaded 89 songs for Eric Clapton, 219 songs for Queen, 783 songs for The Beatles, and 110 for The Rolling Stones. The last dataset we considered consists of classical pieces. We have manually retrieved 64 MIDI files from the following composers: Albanez (24 pieces), Beethoven (29 pieces), and Mozart (21 pieces)

Then we designed a set of deep neural networks, based on transformers, to tackle the classification and generation tasks. We show the results of our networks compared against a set of baselines based on deep learning and machine learning approaches. The high performances of the transformers-based methods confirm their success even in this domain. For the classification task, we show an analysis based on different metrics such as the F1-score, accuracy calculated on the average of 10 iterations through the k-fold cross-validation stratified method. For the generation task, we illustrate the evaluation process used to validate our model through the generated song analysis. We present our results for fifteen different automatically generated songs, knowing that the model to generate these will start from a short excerpt, called seed, of a MIDI file present in the database. For all fifteen songs, we verify their catchiness, their similarity, and their consistency with the song genre from where the seed belongs. To verify the catchiness we present the songs to five people from different musical backgrounds. To verify their consistency with the genre we classify the newly generated songs with the classification model we trained. Lastly, to calculate the similarity between two songs and their vector representation, we used a measure presented in Uitdenbogerd & Zobel (1999), known as the local alignment. This measure was first introduced as a bio-informatics procedure to compare and align two or more amino acid sequences, DNA or RNA. Similar algorithms biologically-inspired have already been used to confront music files (Bountouridis et al., 2017). Through this algorithm, we can confront and know how similar two MIDI files are based on the sequences of equal notes contained in both compositions.

In summary, our contributions can be listed as follows:

• We perform classification at fine and coarse-grained using three different datasets consisting of a small number of tracks.
• We propose two neural networks based on Transformers for the classification and generation of MIDI files.
• We carry out a k-cross validation for the classification task that shows how our proposed neural network transformers-based outperforms the competitors in terms of several metrics such as F1-score and accuracy.
• For the generated songs we perform a manual annotation involving five music experts and an automatic assessment using the local alignment metric indicating the high quality of the generated tracks.

The remainder of this article is organized as follows. In ‘Related Work’ we provide related research on music classification and generation. ‘Background’ provides some information about the MIDI format, how a MIDI file is represented, and what features it contains. ‘Tasks Description’ illustrates the two tasks we tackle in this article. ‘The Adopted Datasets’ describes the datasets we have used. ‘Extraction of Features’ discusses the feature engineering we have performed to create the samples for the used deep neural networks. ‘The proposed deep neural networks’ details them and the transformers we have deployed and used for the aforementioned tasks. ‘Experimental Evaluation’ shows the experiments that we have conducted and analyses the obtained results providing a discussion on them. Finally, ‘Conclusions’ ends the article and illustrates future directions where we are headed.

## Related Work

Music genre recognition and classification are an important part of music information retrieval. They have been deeply studied since 1990. In the first works, authors leveraged signal frequency domain analysis methods (Li & Tzanetakis, 2003). Then, with the advent of machine and deep learning technology, speech recognition and classification have done excellent progresses (Xu et al., 2003). Today, they are both known by the term music genre classification whose goal is the automatic classification of input audio in one of a predefined set of classes. In this work, we deal with MIDI sounds. When dealing with MIDI files, some considerations must be drawn: (i) MIDI files are much more compact than digital audio files, (ii) their size is completely independent of playback quality, (iii) they do not take up as much memory, disk space, or bandwidth, (iv) they can be embedded in web pages load and play more quickly than their digital equivalents, (v) with high-quality samples, they can even create a MIDI mock-up that sounds just as convincing as a real audio recording, (vi) they are completely editable at note level, (vii) they can be converted to musical notation, and vice-versa.

The first consideration to be made is that MIDI music genre classification methods are largely based on generic text classification techniques. As such, Ruppin & Yeshurun (2006) combined techniques of selection and extraction of musically invariant features with classification using compression distance similarity metric. Others combined MIDI files and audio features from MIDI, separately and merged together for MIDI music genre classification using normalized compression distance to compute distances between MIDI pieces (Cataltepe, Yusuf & Sonmez, 2007). For the same task, in Bernardo & Langlois (2008) authors used the pitch levels and durations that describe each track to extract a set of features that are used to train a classifier. Recently, Lisena, Meroño-Peñuela & Troncy (2022) used graph embedding techniques to represent MIDI files as vectors. Basically, MIDI files were represented as a graph and node2vec (Grover & Leskovec, 2016) was then run to generate embeddings using random walks in the graph. The resulting vectors were used for musical genre classification tasks using a Feed-Forward Neural Network.

As far as music generation is concerned, it is defined as the process of composing a short piece of music with minimum human intervention. Examples of MIDI generation are MuseNet (https://openai.com/blog/musenet/), a deep neural network that can create musical compositions lasting four minutes, using ten distinct instruments and combining rock, classical, and country genres. It is able to discover patterns of harmony, rhythm, and style by learning to predict the next token in hundreds of thousands of MIDI files. As MIDI files can be efficiently analysed as a sequence of text, it uses the transformer GPT2 (Radford et al., 2019), well known in the Natural Language and Deep Learning communities to predict the next token in a sequence. Kumar et al. (2022) presented a lightweight deep learning-based method to generate MIDI jazz music. One instrument only was used for the generation. In another contribution (Yang, Chou & Yang, 2017), researchers exploited available prior knowledge, so that the model could generate melodies either from scratch or by conditioning on the melody of previous bars. Moreover, the model could be extended to generate multiple MIDI channels.

Instead of focusing on transformers, Walter et al. (2021) employed generative adversarial networks (GANs) to create music. They adopted the progressive approach towards GANs and implemented it to train on symbolic music data. A user study was finally conducted to evaluate the obtained results, and the Frechet Inception Distance metric was employed.

Donahue et al. (2019) have tuned a model with the LAKH MIDI dataset and then fine-tuned the model on the same NES dataset we have considered in this article. Then they improved the performances of their model by proposing a pre-training technique to leverage the information in a large collection of heterogeneous music. The difference with our work, although, is that we employ a different feature extraction method which completely changes the resulting generated samples.

Other existing deep learning tools for music generation are reported in the following. Magenta (https://magenta.tensorflow.org/music-transformer;  Huang et al., 2019; Hawthorne et al., 2022) is a Google’s open-source deep learning music project that employs a regular recurrent neural network (RNN) and two long short-term memory networks (LSTM’s) to generate music. DeepJazz (https://github.com/jisungk/deepjazz) uses a two-layer LSTM that learns from a MIDI file as its input source and creates jazz music. BachBot (https://github.com/feynmanliang/bachbot/) is a research project at Cambridge University that uses an LSTM to train itself on Bach chorales. Its goal is to generate and harmonize chorales indistinguishable from Bach’s own work. FlowMachines (https://www.flow-machines.com/) is a non-open source system that uses Markov constraints to generate lead sheets based on the style of a composer in a database filled with about 13,000 sheets. WaveNet (https://github.com/ibab/tensorflow-wavenet) is a system designed by researchers at Google’s DeepMind and is based on Convolutional Neural Networks. It uses raw audio as input and it can generate any kind of instrument. GRUV (https://github.com/MattVitelli/GRUV) is a Stanford research project that, similar to Wavenet, employs audio waveforms as input, with an LSTM and gate recorrent unit (GRU) rather than convolutional neural network (CNN).

With respect to the existing state-of-the-art approaches, we perform classification on datasets of different music styles and at fine and coarse-grained levels with the goal to analyse how the classification is affected by the patterns of different genres. Moreover, we designed a deep neural network where we employed the transformer architecture originally proposed in Vaswani et al. (2017) and make use of all the instruments of the input MIDI files for what the feature engineering is concerned. For the generation task, we designed one more neural network where we embedded a transformer architecture known as GPT-2 and proposed in Radford et al. (2019) mixed with the relative global attention mechanism as illustrated in Huang et al. (2018) and implemented in Sigalov (2022). Lastly, to evaluate the generated MIDI sequence we used human annotations for the catchiness of the generated tracks, the fine and coarse-grained classification on the generated songs to check if the predicted classes are compatible with the songs where the seeds have been chosen, and an algorithm called local alignment, introduced in Uitdenbogerd & Zobel (1999), which assesses the quality of the obtained tracks.

## Background

MIDI is a standard protocol developed in the early ’80s and presented for the first time in October 1981 at the Audio Engineering Society in New York.

A MIDI file consists of different blocks of data (chunks). There are two types of chunks:

• **The header chunk**. A MIDI file always begins with the header chunk which contains general information about the MIDI itself. More in detail, the header chunk has the following definition:

\begin{eqnarray*}header\text{_}chunk& =\mathrm{``}MTh{d}^{{^{\prime}}{^{\prime}}}+\lt header\text{_}length\gt +\nonumber\\\displaystyle & \lt format\gt +\lt n\gt +\lt division\gt , \end{eqnarray*}

where the “MThd” is a string of 4 bytes that indicates the beginning of a MIDI file, the *header*_*length* is a 4-byte field that contains the length of the chunk, *format* is a 2-byte field that can specify three different formats (single track file format, multiple track file format, multiple song file format), *n* is 2 bytes and indicates the number of tracks that follow and division is a 2-byte field that represents (if positive) the units per beat.

• **The track chunk**. It stores the notes and contains sequences of time-ordered events (MIDI and non-MIDI). It has the following definition:

\begin{eqnarray*}track\text{_}chunk& =\mathrm{``}MTr{k}^{{^{\prime}}{^{\prime}}}+\lt length\gt +\nonumber\\\displaystyle & \lt track\text{_}event\gt [+\lt track\text{_}event\gt \ldots ], \end{eqnarray*}

where “MTrk” is a string of 4 bytes indicating the start of a track, *length* is 4 bytes and includes the number of bytes in the track chunk following this number, and *track*_*event* is a sequenced track event. Each track_event can represent three types of events: MIDI, SysEx, Meta.

### MIDI events

MIDI events describe how to play the music. The message related to these events has the following structure:

**Table:** 

**Time (ticks)**	**Event type value**	**MIDI Channel**	**Parameter 1**	**Parameter 2**
variable-length	4 bits	4 bits	1 byte	1 byte

According to this structure, each message starts with a timestamp indicating the amount of time to wait before the event starts. It indicates that the event should occur the specified amount of MIDI ticks after the previous event. A MIDI tick lasts an interval time value defined by the time division. The time division of a MIDI file, also known as the “resolution of a MIDI file”, indicates the mapping between a MIDI tick and time in microseconds.

The value of the “resolution of a MIDI file” is obtained from the equation:

(1) \begin{eqnarray*}resolution=(microseconds~per~beat)/(ticks~per~beat)\end{eqnarray*}

The “ticks per beat” value is contained within the division field of the header chunk. The variable “microseconds per beat” may be specified by a META event which we will describe later. Otherwise, it is set to 500,000 microseconds by default. The result indicates how many microseconds a tick should last.^1^ 1For a more detailed description of the “resolution of a MIDI file” please check https://www.recordingblogs.com/wiki/time-division-of-a-midi-file).

After the Time field, it follows a value determining which type of event should start (Event Type Value), and in which of the 16 channels the event should be reproduced (MIDI Channel). The message ends with two optional parameters which contain information for the event such as the note number, or the program number.

There are several possible values of event types contained in the Event Type Value field. In the following, we will report the four most important and pertinent to our study.^2^ 2For a more detailed description of the Standard MIDI-File format please check the following site http://www.music.mcgill.ca/ ich/classes/mumt306/StandardMIDIfileformat.html.

•**Note on**. This type of message describes the moment a note, represented as an integer between 0 and 127, starts playing. The message comes with information about the key velocity (the volume of the note), the channel in which the note will be reproduced, and a timestamp defining how many ticks to wait before the message is executed. A description of the message of type Note on is the following:

**Table:** 

**Message**	**Time (ticks)**	**Channel**	**Note Number**	**Velocity**
NOTE ON	t	0–15	0–127	0–127

•**Note off**. Similarly to Note on, it defines when a note, in a channel, should stop playing. In the following a description of this type of message is reported:

**Table:** 

**Message**	**Time (ticks)**	**Channel**	**Note Number**	**Velocity**
NOTE OFF	t	0–15	0–127	0–127

•**Program Change**. In the MIDI language, a program is a synonym for an instrument used to play the note. This event is used to select the instrument that will play the notes in a channel. A structure of such a type of message is described in Table 1.

**Table 1:** Structure of the program change message.

**Message**	**Time (ticks)**	**Channel**	**Program Number**
PROGRAM CHANGE	t	0–15	0–127

•**Pitch Bend**. This event is used to change the pitch value of a MIDI Channel. The pitch value affects all the notes on the current message. A structure of such a type of message is reported in Table 2.

**Table 2:** Structure of the Pitch Bend message.

**Message**	**Time (ticks)**	**Channel**	**LSB**	**MSB**
PITCH BEND	t	0–15	0–127	0–127

### SysEx events

System-exclusive events are the second category of event (SysEx) and are employed to command MIDI hardware or software that needs unique data in accordance with the manufacturer’s instructions. An ID is included in every SysEx event that identifies the manufacturer’s product that is the intended recipient. Three types of SysEx messages (Normal, Divided, and Authorization), are used to deliver data in a single event, across many events, or to authorize the transmission of particular MIDI messages.

### META events

The third, and last, type of messages that can be included in the track_event is the META events. These are also known as non-MIDI events. Usually, they send additional information about the MIDI file, such as text, copyright, sequence/track name, instrument name, lyrics, and so on. One important type of META event is the MIDI set tempo. This event sets the tempo of a MIDI sequence in terms of microseconds per quarter note defining the parameter value for the resolution of the MIDI file as indicated in Eq. (1).

## Tasks Description

In this article, we tackle two tasks for MIDI songs: classification and generation.

### Classification

The classification task we faced is multi-class single-label, that is each MIDI file can be associated with one class only (that is why single-label) among a list of different classes to choose from (that is why multi-class). We perform two types of classification: one fine-grained and the other coarse-grained. The reason to have defined the two types of classification is to understand whether MIDI patterns from different composers of the same genre are more difficult to recognize than MIDI patterns from different genres. Therefore, for the first type of classification we analyse (fine-grained), we perform a separate classification on every single dataset we have collected to identify the class or author of each track in the test set. In this case, the task is intuitively harder because the classifier will have to detect peculiarities within MIDIs of the same genre (*i.e.,* identifying the authors of different rock songs). The other classification (coarse-grained) is performed on the combination of all the datasets we have collected where each dataset corresponds to a different class to be identified. In such a case the classification should be easier as the MIDI patterns should be more similar among tracks of the same dataset and more different between tracks of different datasets (*i.e.,* identifying whether a certain song is a rock or classical or other musical genres).

### MIDI Generation

Text generation is a task in computational linguistics whose aim is to generate natural language texts which can be correct both syntactically and semantically. We have mapped the MIDI generation as a text generation task where the generated notes should be consistent with the input seed. The idea is to treat a MIDI track as a simple text, with tokens for note values, note duration, and for time, which denotes when the related note should start playing.

We have therefore used a language model provided by the transformer-based neural network architecture (in ‘The proposed deep neural networks’ we will provide further details about that). The generated music sequences are the result of the completion of selected short initial parts of music files in the database, with which the network has been trained. This sequence is completed in order to generate a MIDI with a suitable structure, and consistency with the track from which the short initial part is extracted. In presence of a small training set, the generated sequences have higher chances to be more similar to the ones used as a seed, so the resulting MIDI can be a revisitation of the seed and needs to maintain consistency and structure, while at the same time being original respect to the sequence from where the initial part was extracted.

## The Adopted Datasets

In this section, we will describe the datasets we have adopted for classification and generation.

### Nintendo Entertainment System (NES) database

The NES dataset has been introduced in Donahue, Mao & McAuley (2018). It is intended for building automatic music composition systems for the NES audio synthesizer. It contains 5278 songs from the soundtrack of 397 NES games.

The database can be considered a structurally homogeneous collection of MIDI files based on the song type it contains. The type is called “Chiptune“ or “8-bit“, which is firmly connected to the history of video games, and its style is strongly influenced by the sound limitations that the video games of the late eighties, and early nineties, posed to developers and composers. This origin gives the genre unique features, recognizable by iconic synthesized sounds traditionally made using a “Programmable Sound Generator“ (PSG), an integrated circuit capable of generating sound waves and synthesizing different waveforms. Although the Chiptune can interpret countless musical genres, its timbre is immediately recognizable. From Super Mario and Castlevania to Shovel Knight and Fez, “Chiptune“ music is still alive to this day.

The 5,278 songs include all the music from a specific video game, from long theme songs to short sound effects played when a certain action takes place in the game (for instance, a short game over sound). In our article, we decided to exclude the MIDI files containing less than 128 note events, keeping for our purposes only the long theme songs. The number of MIDI files with less than 128 note events was 1,631. The remaining 3,636 MIDI files (which correspond to 397 resulting NES games) have been divided into chunks of the exact length of 128 melody chords, each one containing four integers (channel velocity, time, duration, pitch). Thus, a chunk after the post-processing is 512 tokens long and a song can be subdivided into a different number of chunks. In the following, we will show some statistics from these 3,636 MIDI files. In particular, Table 3 shows the average duration of the NES MIDIs, their overall duration, the standard deviation of their duration, and the minimum and maximum duration.

**Table 3:** Statistics on NES soundtracks length.

**Average Duration**	**Total Duration**	**Duration Standard deviation**
49 s	140,253 s	57 s

**Minimum duration**	**Maximum duration**
2 s	1,517 s

We identified five main categories of NES video games: Role Playing Games (RPG), Sports, Fighting, Shooting, and Puzzle. Three annotators (players of old video games) manually labeled 1,728 of the 3,636 NES soundtracks based on the main category of the video games they came from. Each MIDI was associated with one of the five classes. There were never cases of disagreement. Table 4 illustrates the number of MIDIs per category.

**Table 4:** Number of MIDI tracks per NES videogame genre.

**RPG**	**Sports**	**Fighting**	**Shooting**	**Puzzle**
746	161	218	443	242

**Figure 1:**
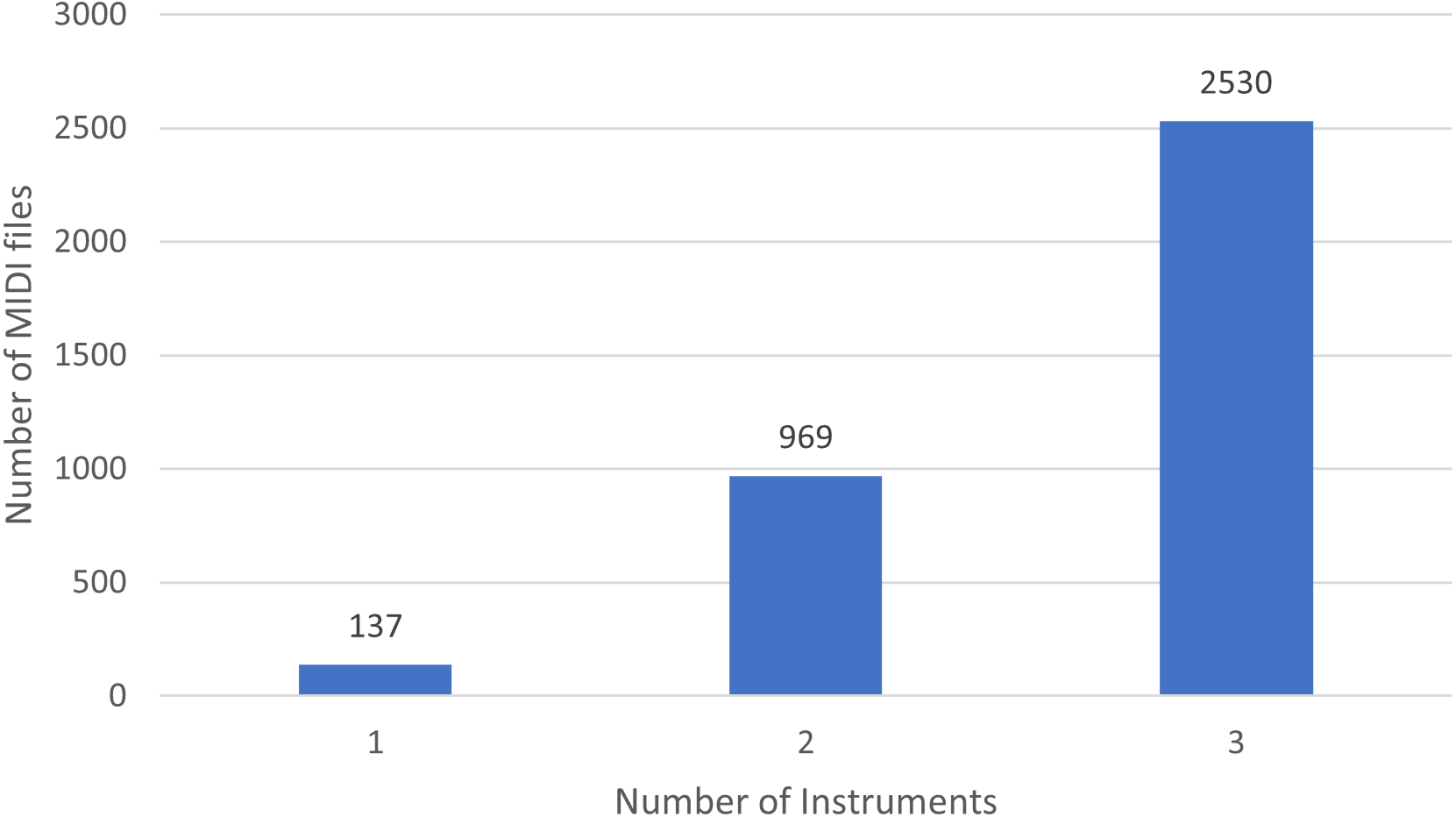
Distribution of instruments in NES MIDI files.

Moreover, in Fig. 1 we show the number of MIDI files split by the number of played instruments. Basically, there are 137 soundtracks of NES games with one single instrument, 969 with 2 different instruments, and 2,530 with three.

**Figure 2:**
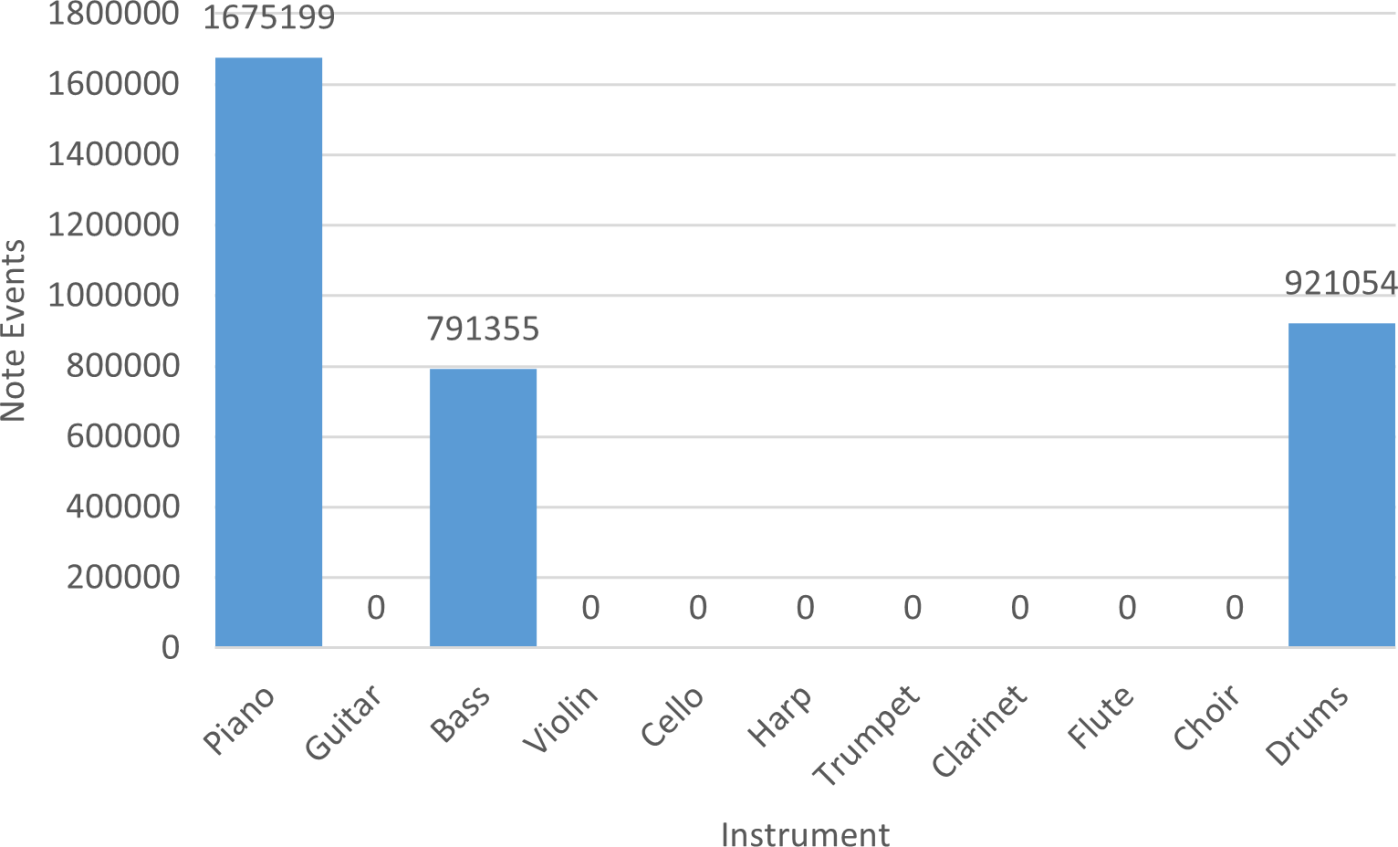
Number of note events per instrument in the NES dataset.

Finally, in Fig. 2 we indicate the number of note events of the entire collection with respect to each instrument. Basically, piano, bass, and drums are recurrent instruments in NES soundtracks.

### Rock MIDI database

We created a small collection of instrumental rock MIDIs from four different artists for a total of 1,199 songs, divided as indicated in Table 5. MIDI files have been extracted from PianoMidi (http://www.piano-midi.de/) and MAESTRO (https://magenta.tensorflow.org/datasets/maestro).

**Table 5:** Number of MIDI files per rock artist.

**The Beatles**	**Queen**	**Eric Clapton**	**The Rolling Stones**
782	218	89	110

In the following, we will show some statistics from these 1,199 songs. In particular, Table 6 shows the average duration of the rock MIDIs, their overall duration, the standard deviation of their duration, and the minimum and maximum duration.

**Table 6:** Statistics on rock songs length.

**Average Duration**	**Total Duration**	**Duration Standard deviation**
230 s	274,132 s	583 s

**Minimum Duration**	**Maximum Duration**
22 s	19,652 s

**Figure 3:**
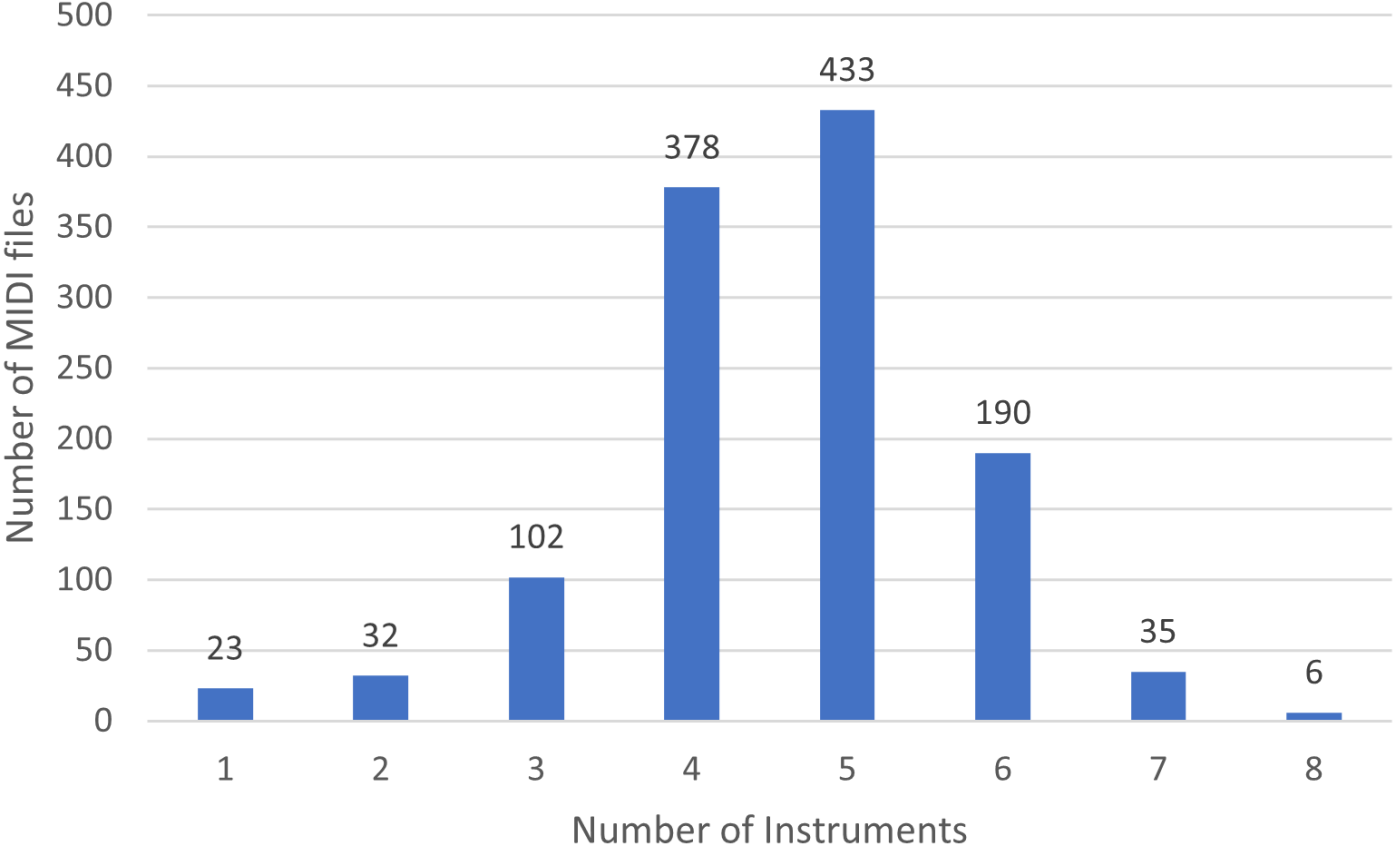
Distribution of instruments in the rock dataset.

In Fig. 3 we show the number of MIDI files split by the number of played instruments.

**Figure 4:**
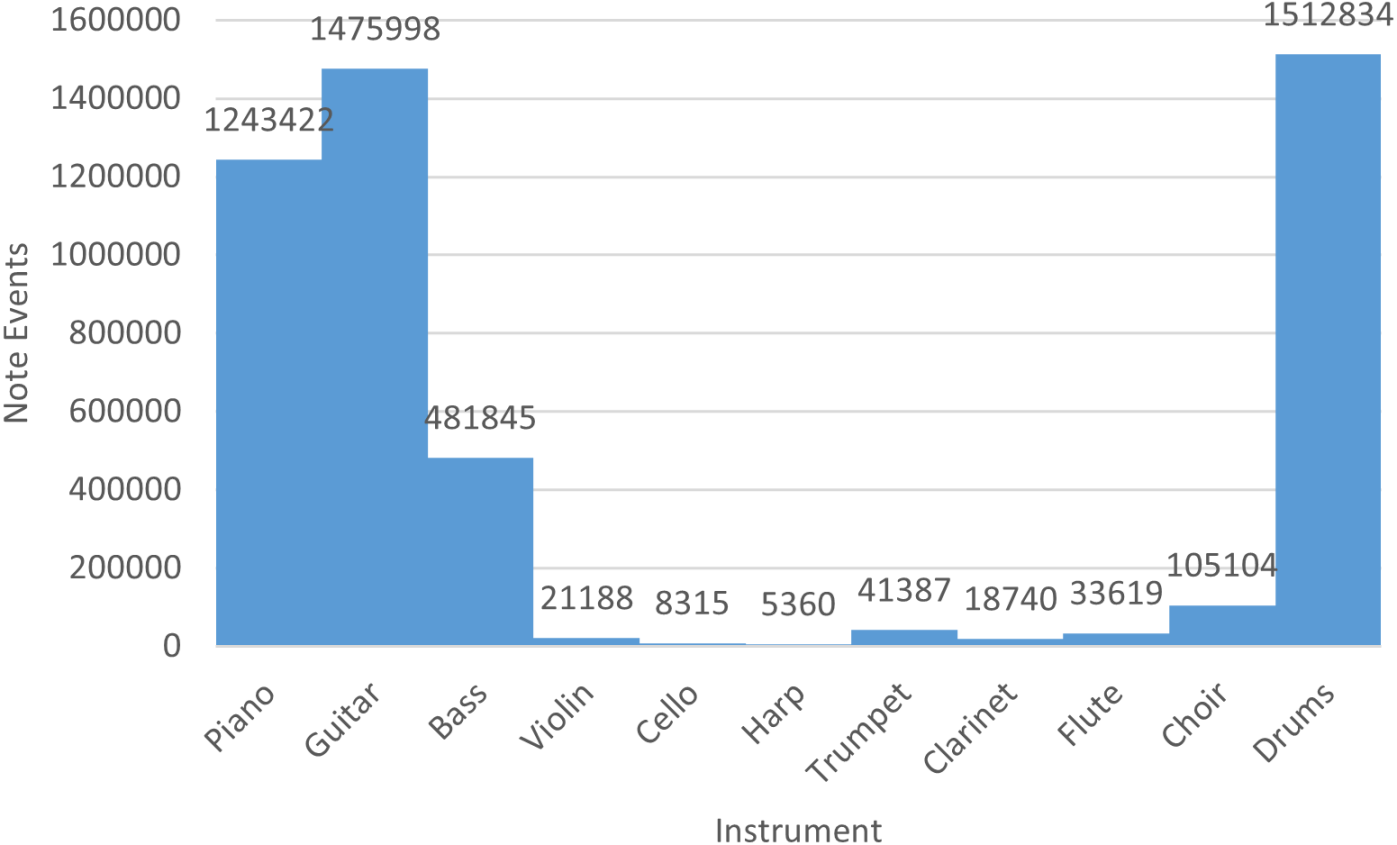
Number of note events per instrument in the rock dataset.

Finally, in Fig. 4 we indicate the number of note events of the rock collection with respect to each instrument. Basically, piano, guitar, bass, and drums are recurrent instruments of the rock MIDI dataset.

### Classical MIDI database

Finally, we created a small collection of classical music MIDI files from three different composers for a total of 101 songs, divided as illustrated in Table 7. MIDI files have been extracted from Kunstderfuge (https://www.kunstderfuge.com/) and ClassicalArchives (https://www.classicalarchives.com/midi.html).

**Table 7:** Number of MIDI files per classical composer.

**Albanex**	**Beethoven**	**Mozart**
28	42	31

Table 8 shows the average duration of the classical MIDIs, their overall duration, the standard deviation of their duration, and the minimum and maximum duration.

**Table 8:** Statistics on classical songs length.

**Average Duration**	**Total Duration**	**Duration Standard deviation**
384 s	24,632 s	271 s

**Minimum Duration**	**Maximum Duration**
95 s	2,011 s

The reader notices that all the note events in these MIDI files are associated with the piano. There are a total of 330,012 note events.

## Extraction of Features

We have used the library TMIDIX (https://asigalov61.github.io/tegridy-tools/TMIDIX.html) to handle MIDI files. The library is able to read and create these files and has all the functionalities to extract the information previously illustrated. The TMIDIX library provides functions that create a structure called *opus*, which contains the sequence of MIDI events for each track in the file and the “resolution of the MIDI”. We are only interested in extracting this type of events since they specify the musical instructions. In Table 9 the structure of *opus* is shown:

**Table 9:** Structure of opus.

**Time (ticks)**	**Tracks #**	**Optional Tracks**
Integer	Events List	Events List
	(Program Change, Note On, Note Off)	

An example of an opus value is the following:

 
 
[ 96, [ # track 0: 
    [‘program_change’, 0, 1, 8], 
    # and these are the events 
    [‘note_on’, 5, 1, 25, 96], 
    [‘note_off’, 96, 1, 25, 0], 
    [‘note_on’, 0, 1, 29, 96], 
    [‘note_off’, 96, 1, 29, 0], 
    ], # end of track 0 
]    

Time values (0, 5, 96, 0, 96) indicate the MIDI ticks that each related event should wait to occur after the previous event. Meanwhile, the first 96 value indicates the number of ticks per quarter in this example MIDI.

An important step is to transform all the time values previously mentioned from ticks to milliseconds (or microseconds). We consider a base value of 400 ms per quarter for each processed MIDI; in this way, we obtain the milliseconds’ value for each tick (in this case 400/96 = 4,16). With this value, we recalculate the other times. For instance, since the first ‘Note On’ message has five ticks, and since each tick is equal to 4,16 ms, its new timing will be 5*4,16 = 20,8 (21 rounded). In order to perform this task, we map all the time (or ticks) values in the *opus* structure to millisecond values obtaining the following structure:

 
 
[1000, [# track 0: 
    [‘set_tempo’, 0, 1000000], 
    [‘program_change’, 0, 1, 8], 
    [‘note_on’, 21, 1, 25, 96], 
    [‘note_off’, 400, 1, 25, 0], 
    [‘note_on’, 0, 1, 29, 96], 
    [‘note_off’, 400, 1, 29, 0]]]    

Here, we added the set_tempo event to further redefine the times’ value to transform one tick in one millisecond. The set_tempo event resets the value of microseconds per beat to 1,000,000 and the first value in the structure (1,000) represents the division value from the MIDI header. These values will be used to calculate the correct tick as seen in Eq. (1). According to the equation, we obtain 1,000,000/1000 = 1,000 ms; therefore, if we convert the results, one tick will correspond to 1,000 ms, which is equal to 1 millisecond.

The resulting *opus* structure is then transformed into another format, called *score*. The score format differs from the *opus* format for the following characteristics:

• Times are expressed with an absolute number of ticks calculated from the beginning of the track. For instance, we can observe the second ‘Note’ event that now has 421 as a time value and not 400 as before.
• The pair of “Note On” and “Note Off” events in the *opus* format are combined into a single note event with the following structure:

[*’note’, start time, duration, channel, pitch, velocity* ]

The score value obtained from the previous opus structure is the following:

 
 
[1000, [# Track 0 
    [‘set_tempo’, 0, 1000000], 
    [‘program_change’, 0, 1, 8], 
    [‘note’, 21, 400, 1, 25, 96], 
    [‘note’, 421, 400, 1, 29, 96]]] 
]    

The *score* format will be further processed to obtain the *melody chord* format where we will have a new representation of the note events and we will discard all the other event types different than the “Program change”.

From the “Program change” event we extract the channel (1 in the example) and the program number (8 in the example) that set which channel and which instrument that follows this message will be used for playing. We compare the extracted instrument number with a list of default instruments set in the TMIDIX library: piano, guitar, bass, violin, cello, harp, trumpet, clarinet, flute, and choir. If a match is found, then the note channel value is set to the index of the matched instrument, otherwise, the note channel value is set to a default value.

If a note has a channel value higher than 9 (the first channel value is 0), the note is discarded for the sake of simplicity as the dimension of the instruments defined within the TMIDX library is 10. After these steps, we obtain an intermediate representation having the following form:

[ *Event* *type*, *Start* *Time*, *Duration*, *Channel*, *Pitch*, *Velocity*, *Program* *Number*]

From the previous score format we therefore obtain:

[[’*note*’, 21, 400, 0, 25, 96, 8, ’*note*’, 421, 400, 0, 29, 96, 8]]

From here we recalculate the timings (start time and duration) dividing them by 16 and 32 as a normalization step and obtaining, respectively, 1 as the new start time (21/16) and 26 (421/16). For the duration, we divide by 32 and obtain 12 (400/32) for both the note events:

[[’*note*’, 1, 12, 0, 25, 96, 8, ’*note*’, 26, 12, 0, 29, 96, 8]]

We do not want to process songs with too few note events since they can add noise to the training phase and are not representative. Thus, in this project, we keep songs with at least 128 note events.

The *melody chord* has the following structure:

*Melody  Chord = [Channel  velocity, Time, Duration, Pitch]*

Channel velocity combines the information of the channel representing the instrument that should play and its velocity, multiplying the channel value by 10 and adding the velocity divided by 16 for each note event. In the previous example, taking into account the score structure explained before, the channel velocity becomes 6, corresponding to (0 * 10) + (96/16).

The time represents the moment when the note should start playing and is obtained from the difference between the current note event time we are processing and the time from the previous note event plus 128. The duration of the note is the number of ticks that should last and corresponds to the known duration value present in the opus structure plus 256. The pitch value is obtained from the last pitch value plus 384.

The summation by 256 and 384 is intended to be a normalization technique allowing us to scale all the values in the melody chords between 0 to 512.

From the previous *opus* structure we obtain the following *melody chords*, where each list represents a note event: 3Here the previous note event is considered to have time 1.

*Melody Chords* = [[6, 128^3^, 268, 409], [6, 153, 268, 413]]

Lastly, we decompose the melody chords representation to have a single list of integers representing the features extracted from the MIDI as follows:

[6, 128, 268, 409, 6, 153, 268, 413]

Thus, a certain song will consist of different note events where each one will be represented by four numbers. The neural nets we have designed and all the used baseline methods will receive as input this integer representation for a total length of 512 integer tokens in each sample for the classification and 1,024 for the generation, with values in a range between 0 and 512.

## The Proposed Deep Neural Networks

### The developed network for classification

**Figure 5:**
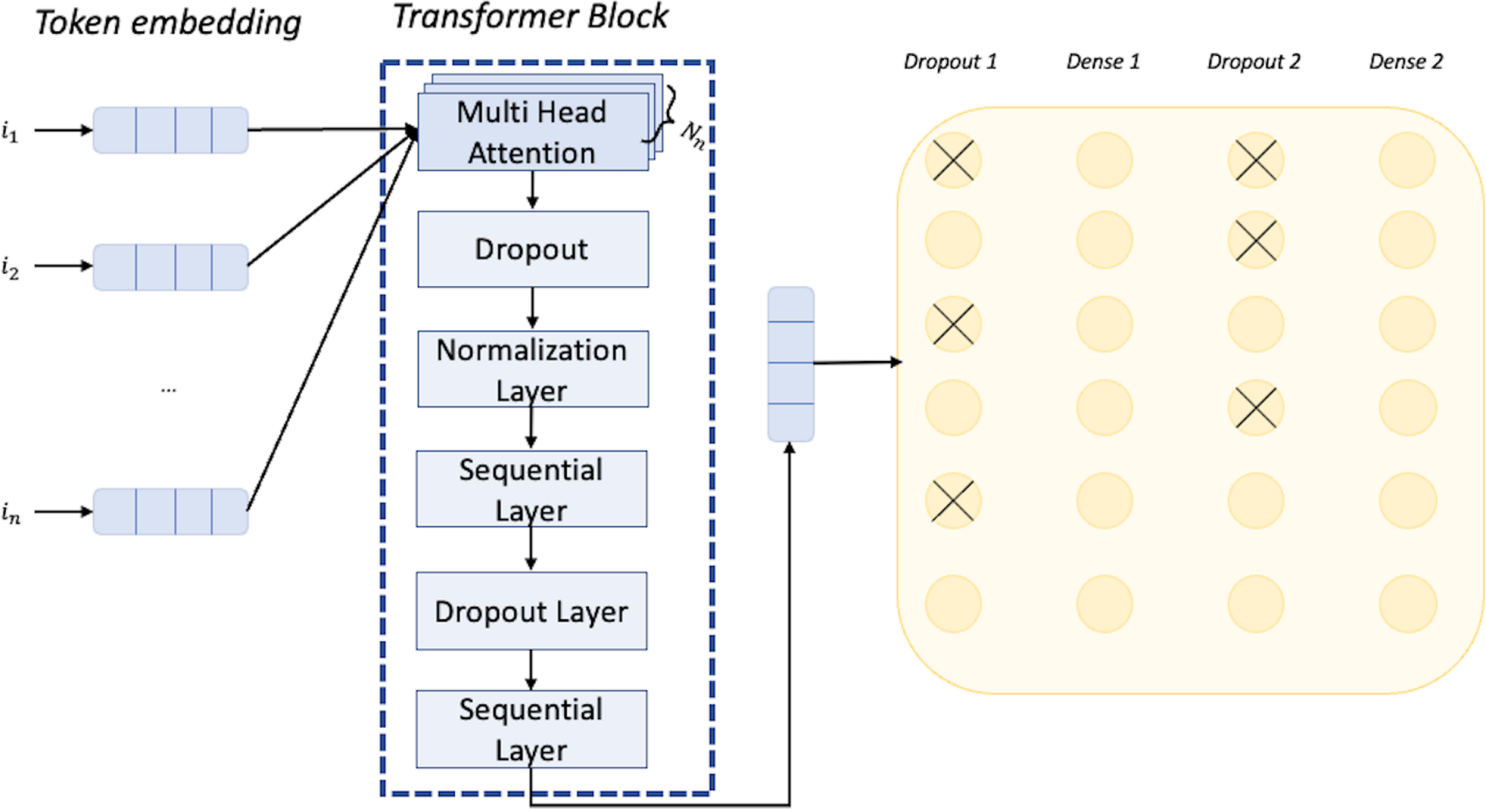
Architecture of the neural network used for the classification task.

The network we have employed is based on a transformer architecture. The first layer of the network is the input layer that can receive data. Specifically, in our case, the input will be a MIDI music excerpt, represented as a sequence of tokens. Each token can have one of 512 different values corresponding to the dictionary size. Next, we have the token and position embedding layer with an embedding dimension of 256. This layer encodes position information, allowing the network to understand the temporal structure of the music sequence. This is particularly important for music, where the order and timing of notes and events are crucial to the overall sound and feeling of the piece. The encoded vector is taken in input by the transformer block. At the start of the transformer block we have the multi-head attention layer, which enables the model to combine knowledge from different dependencies, being especially useful for dealing with long-term dependencies in music. Specifically, we select eight heads, meaning that the layer produces eight different outputs capturing different aspects of the input sequence (*e.g.*, long-range *vs* short-range). The result of each head is concatenated, and using a feed-forward with a dimension of 64, we apply a linear transformation to the concatenated result, to produce a single representation of the sequence. This representation is the output of the transformer block that is passed to a dropout layer that randomly drops out some of the values, which helps to prevent overfitting by forcing the model to learn more robust representations. Then we have a dense layer applying a ReLu activation function to the output of the dropout layer, which further introduces non-linearity to the model. The output is passed to another dropout layer and in the end, reaches a final dense layer with a softmax as an activation function. The softmax function converts the output of the previous layer into a vector of dimension K, in which K are the different classes. Each element of the vector will contain the probability of the input sequence belonging to the particular class. During training, the network adjusts the weights and biases of the dense layer to minimize the implemented categorical cross-entropy loss between the predicted probability distribution and the true label distribution for each MIDI music excerpt. The cross-entropy loss measures the dissimilarity between the predicted probability distribution and the true label distribution. We set the other important hyperparameter value of the learning rate to have a rate decay factor based on the formula (final learning rate/initial learning rate)∗(1/*epochs*) with final learning rate value equal to 0.000001, initial learning rate value equal to 0.0001, and a number of epochs equal to 20. The architecture of the network we employed for classification is shown in Fig. 5. For the development of such a network, we used the Keras framework (https://keras.io/).

### The developed network for generation

The network we employed for the generation task is based on transformer technology. In particular, we made use of the GPT-2 (Generative Pretrained transformer) architecture mixed with the Relative Global Attention (RGA) as implemented in Sigalov (2022), from where we downloaded the pre-trained model that was trained on the Full LAKH MIDI dataset converted to a MIDI output format (nine instruments + drums) (https://github.com/asigalov61/LAKH-MuseNet-MIDI-Dataset) Raffel (2016). The used format is coherent to what we discussed in ‘Extraction of Features’.

More precisely, the pre-trained model was trained with 260.000 MIDI excerpts for about 44 h on dual A6000 GPUs with 28 as batch value.

The self-attention mechanism of the original transformer does not explicitly model any positional information in the input data, so the RGA (also called Relative Position Representations), implemented in the pre-trained model we presented below, extends the self-attention mechanism to efficiently consider this positional information.

The RGA mechanism, introduced in Shaw, Uszkoreit & Vaswani (2018), extends the self-attention mechanism to model relative positions between tokens in the input sequence. It does this by introducing additional information into the attention calculation, specifically the relative position of each token with respect to a fixed reference point. This allows the model to capture more nuanced relationships between tokens in the input sequence. To use RGA, each token in the input sequence is associated with a relative position embedding, which encodes the distance between that token and a reference point. The reference point can be any token in the input sequence, but in practice, it is often chosen to be the first or last token (in our model the reference is the first token). These relative position embeddings are then used in the attention calculation, in addition to the standard self-attention scores. In the context of generating MIDI music, using RGA instead of standard self-attention may help the model to capture more complex relationships between notes, such as patterns or motifs that repeat at different points in the sequence. This could lead to a more sophisticated and interesting music generation.

**Figure 6:**
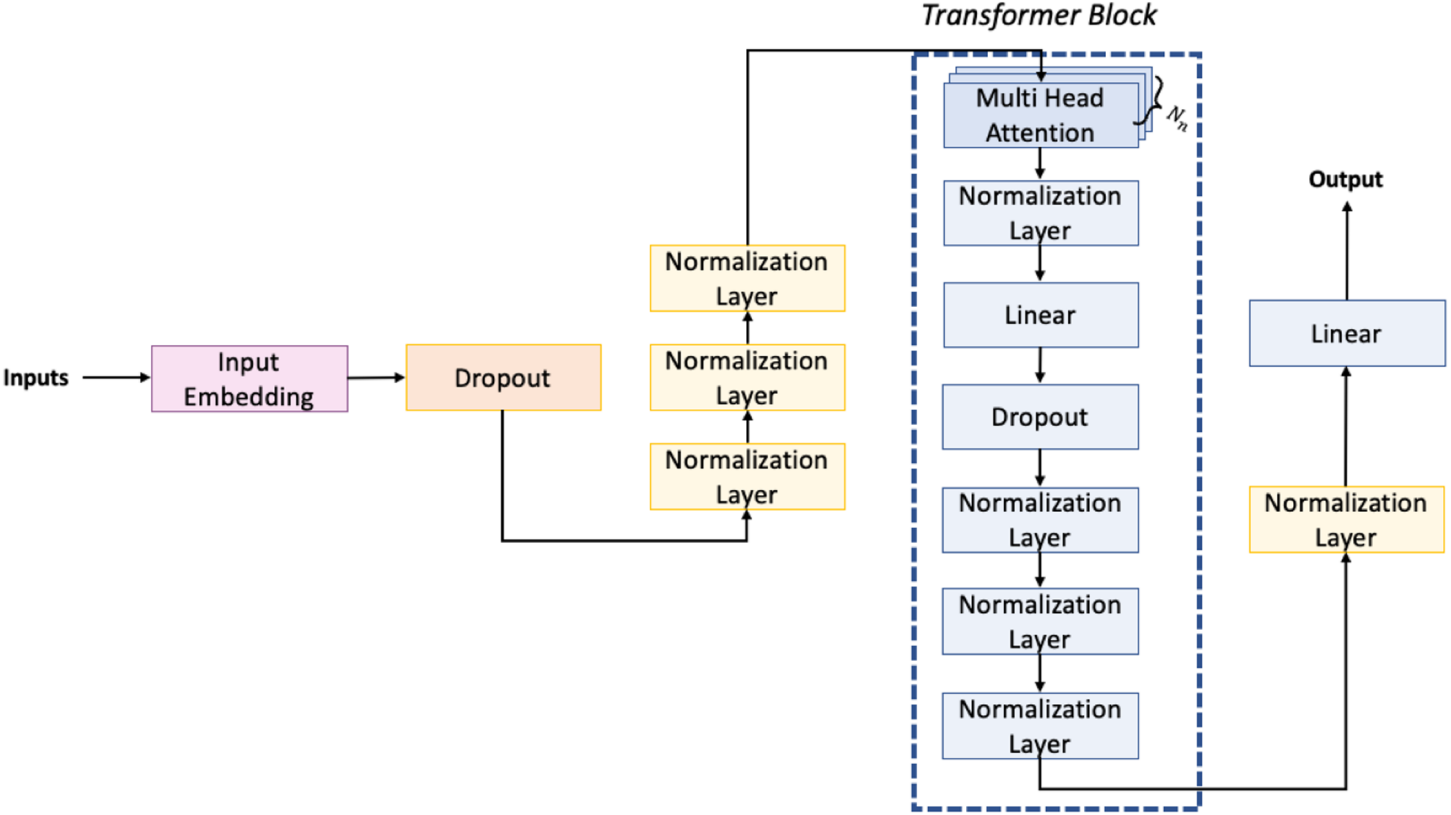
Architecture of the neural network used for the generation task.

The architecture of the network we have used is shown in Fig. 6. The first embedding layer takes as input data 1,024 tokens as the maximum dimension and, since each token may have one of 512 different values, we use for each of them an embedding dimension of 512, defined by the output shape of the embedding layer (1,024, 512).

Then we have the dropout layer, which is necessary to prevent the overfitting of the neural network. Usually, the dropout layers randomly set a portion of the input to zero. Specifically, in our neural network this portion is set to 10% of the input units. Next, to improve the stability and performance of the model, we have inserted three normalization layers. Then, in the architecture, we can observe the presence of transformer blocks. These blocks are the main component of transformer-based neural networks. Each transformer block consists of several layers, including multi-head attention, normalization, feedforward, and dropout. The multi-head attention layer allows the network to attend to different parts of the input sequence simultaneously and has inside a feedforward layer that applies a nonlinear transformation to each input element. In our network, we have eight transformer blocks, each with an output dimension of 512 for the linear layers. The final layer of the network is a dense layer with a softmax activation function. This layer takes the output of the last transformer block and maps it to a probability distribution over the possible next tokens, selecting the one with the highest probability score. Overall, our model is designed to take a sequence of up to 1024 tokens and generate a probability distribution over the possible next tokens. The use of transformer blocks allows the network to capture complex patterns in the input sequence, while the normalization layers and dropout layer help to prevent overfitting and improve performance.

For the fine-tuning, the input songs, processed as seen in ‘Extraction of Features’, have been given to the network in chunks of a max length of 512 integer tokens with a batch size of 4. Note that although the network can handle sequences of up to 1,024 tokens, we use 512 as length value for fine-tuning on our dataset due to computational limitations. We trained for only one epoch for each dataset due to the time-consuming task; within this time the network weights, already trained on the LAKH MIDI, should be able to change according to the dataset we are fine-tuning on, specializing each time towards a different music genre.

## Experimental Evaluation

In this Section, we will illustrate the evaluation procedure we have carried out. First, in ‘Results on Classification’ we will show the results we have obtained for the classification, and then in ‘Results on Generation’ those obtained for the generation.

### Results on classification

As previously mentioned, the classification task has been carried out at fine and coarse-grained levels. The former was performed on each of the three dataset: NES, rock, and classical. The conducted experiments are based on the comparison of the results obtained by our proposed neural network with other machine learning baselines (k-nearest neighbour, random forest, and support vector machines) and other deep learning approaches (Long short-term memory LSTM Jing (2019) and Convolutional Neural Networks CNN (https://cezannec.github.io/CNN_Text_Classification/)). The strategy used to measure the system performances is the k-fold cross-validation stratified, implemented through SciKit Learn (https://scikit-learn.org/stable/). The k-fold technique allows us to accurately validate the performance of our model by dividing the dataset at each iteration into the following non-overlapping chunks: test set (twenty percent of the dataset), training set (eighty percent of the dataset). The training set is further split into the actual data used for training (90 percent) and a validation set (10 percent)

#### NES dataset

In the NES dataset, we extracted a total of 1728 labeled tracks. Each track is represented by different arrays (also referred to as samples) of a fixed length of 512 tokens (integer) for a total of 13.473 samples. If the last sample of a certain track is less than 512 tokens then it is padded with 0s.^4^ 4The padding is performed also for the classifications on the other datasets.Considering the class of the original track, the resulting label distribution of these samples is shown in Table 10.

**Table 10:** Classes distribution of the samples for the NES dataset.

**RPG**	**Sports**	**Fighting**	**Shooting**	**Puzzle**
5,251	750	1,604	4,397	1,471

At each iteration of the k-fold cross-validation, we have 9,700 samples for the training set, 1,078 for the validation set, 2,695 for the test set. The sets are created stratified, meaning that we preserve the same proportions of samples in each class as observed in the original dataset and for each set. This observation holds for all the other classification experiments we have carried out at fine and coarse-grained levels.

**Figure 7:**
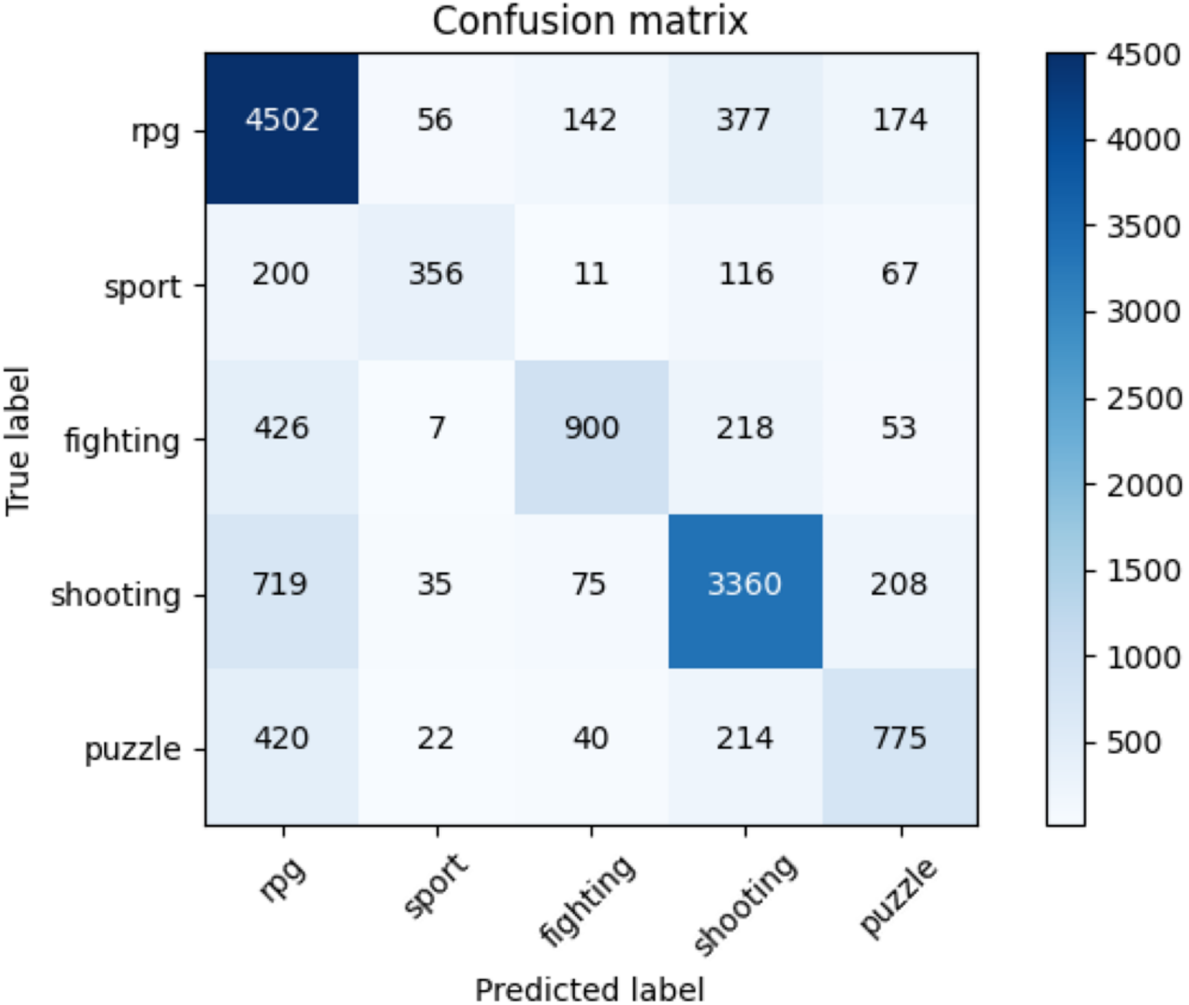
Confusion matrix summed across all the folds for the NES dataset using the adopted transformers model.

Table 11 indicates the average accuracy score and the ROC AUC score whereas Table 12 illustrates the F1-score for each class of NES dataset and each adopted method. The reader can notice the higher performances of the transformers against the baseline approaches. Moreover, for the transformer approach, we indicate in Fig. 7 the confusion matrix including the summed values across all the folds.

**Table 11:** Classification results in terms of accuracy and ROC AUC score on the NES dataset averaged over all the classes.

	**Accuracy score**	**ROC AUC score**
Nearest Neighbour	0.40	0.53
Random Forest	0.42	0.54
Support Vector Machine	0.26	0.50
LSTM	0.65	0.68
CNN	0.62	0.65
Transformer-based	0.73	0.78

**Table 12:** Classification results in terms of F1-score on the NES dataset. NN stands for nearest neighbor, RF for random forest, SVM for support vector machine and T-based for transformer-based.

	**RPG**	**Sport**	**Fighting**	**Shooting**	**Puzzle**	**Macro Average**	**Weighted Average**
NN	0.45	0.008	0.06	0.49	0.05	0.21	0.34
RF	0.42	0.004	0.002	0.34	0.01	0.15	0.27
SVM	0.35	0.08	0.16	0.29	0.12	0.20	0.27
LSTM	0.72	0.62	0.64	0.74	0.60	0.65	0.72
CNN	0.70	0.61	0.59	0.71	0.58	0.61	0.67
T-based	0.78	0.64	0.69	0.79	0.60	0.70	0.75

In Table 12, we present the results (in terms of F1-score) of the k-cross-validation for each class, for the baseline methods we implemented, and the adopted solution using the transformer we employed. The reader can notice how our approach outperforms all the other machine learning baselines on each class of the NES dataset and on average (at macro and weighted levels).

#### Rock dataset

In the rock dataset, we have a total of 1,199 labeled tracks. Each track is represented by different arrays of a fixed length of 512 integer tokens for a total of 40,120 samples. Considering the classes of the original track, the resulting label distribution of these samples is shown in Table 13.

**Table 13:** Classes distribution of the samples for the rock dataset.

**Eric Clapton**	**Queen**	**The Beatles**	**the Rolling Stones**
3,839	7,799	23,123	5,359

At each iteration of the k-fold cross-validation we have 25,676 samples for the training set, 6,420 for the validation set, and 8,024 for the test set.

**Figure 8:**
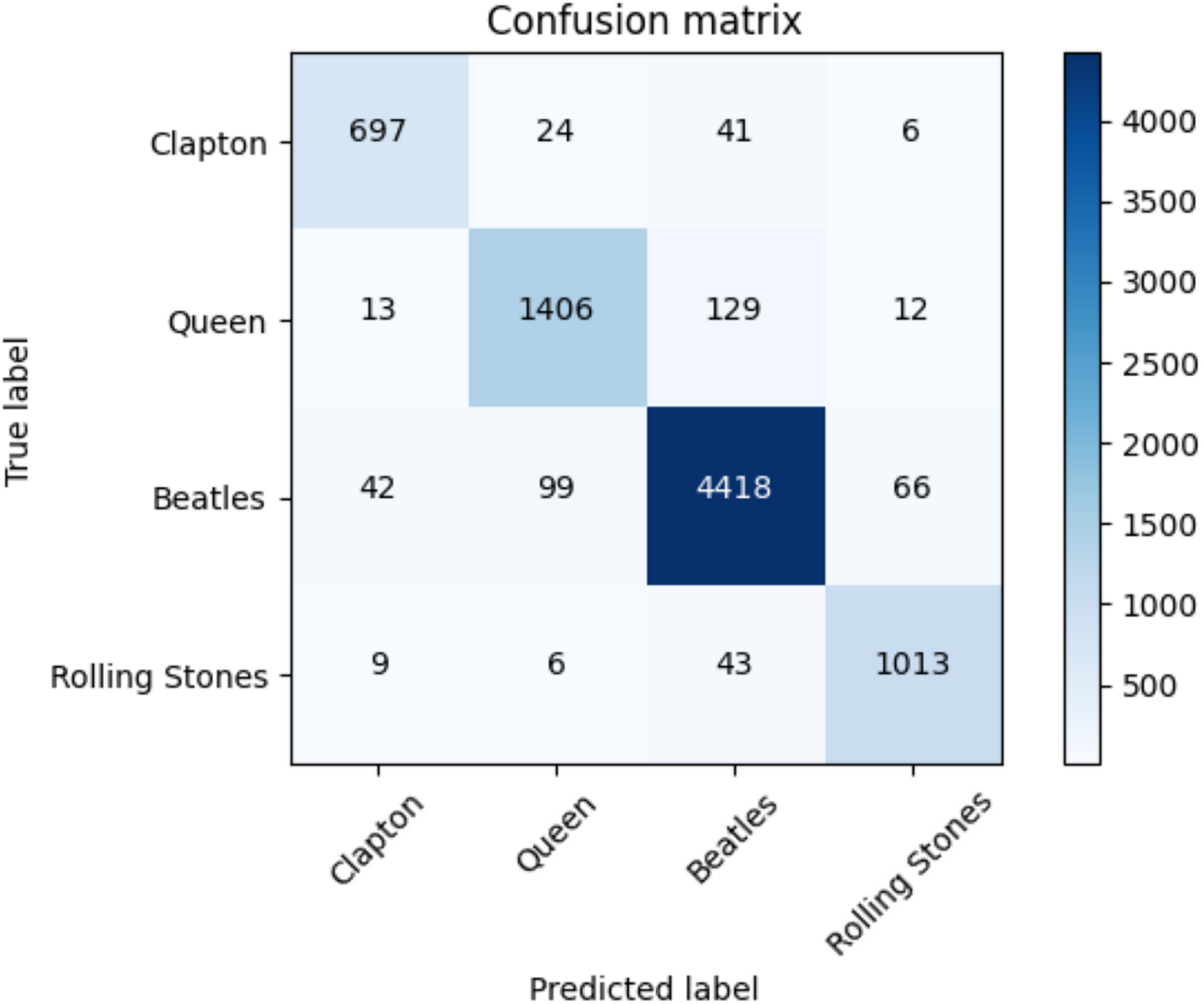
Confusion matrix summed across all the folds for the Rock dataset using the adopted transformers model.

The average accuracy score and the ROC AUC score are depicted in Table 14. The F1-score for each class of the Rock dataset and each adopted method is reported in Table 15. The performances of the transformers are higher than those of the baseline approaches. Figure 8, moreover, indicates the confusion matrix of the summed values across all the folds for the classification performed with the transformer.

**Table 14:** Classification results in terms of accuracy and ROC AUC score on the rock dataset averaged over all the classes.

	**Accuracy score**	**ROC AUC score**
Nearest Neighbour	0.54	0.57
Random Forest	0.61	0.63
Support Vector Machine	0.52	0.50
LSTM	0.83	0.86
CNN	0.87	0.89
Transformer-based	0.94	0.95

**Table 15:** Classification results in terms of F1-score on the rock dataset.

	**Eric Clapton**	**Queen**	**The Beatles**	**The Rolling Stones**	**Macro Average**	**Weighted Average**
Nearest Neighbour	0.16	0.32	0.69	0.23	0.35	0.51
Random Forest	0.11	0.38	0.79	0.43	0.43	0.60
Support Vector Machine	0.12	0.27	0.47	0.15	0.25	0.36
LSTM	0.89	0.90	0.92	0.92	0.91	0.92
CNN	0.87	0.86	0.90	0.90	0.89	0.91
Transformer-based	0.92	0.92	0.96	0.94	0.93	0.94

Results of F1-score of the k-cross validation are indicated in Table 15. Each class is reported together with the F1-score of each baseline method and our proposed solution. The reader can observe how our transformer-based approach outperforms all the other baselines in each class and on average (at macro and weighted levels).

#### Classical dataset

In the classical dataset we have a total of 101 labeled tracks. Each track is represented by different arrays of a fixed length of 512 integer tokens for a total of 2630 samples. Considering the class of the original track, the resulting label distribution of these samples is shown in Table 16.

**Table 16:** Classes distribution of the samples of the classical dataset.

**Albanez**	**Beethoven**	**Mozart**
414	1,293	923

At each iteration of the k-fold cross-validation we have 1,893 samples for the training set, 211 for the validation set, 526 for the test set.

**Figure 9:**
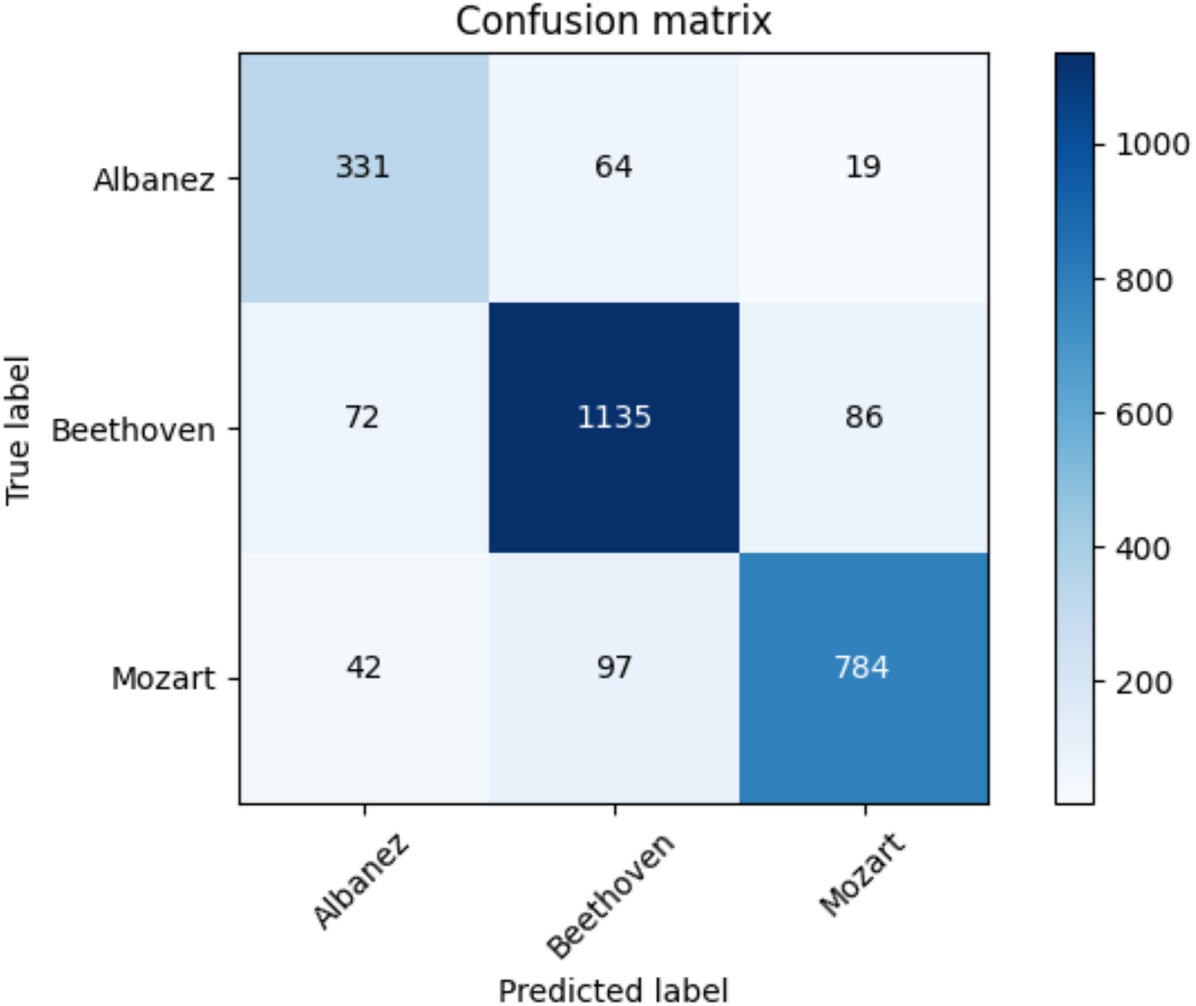
Confusion matrix summed across all the folds for the classical dataset using the adopted transformers model.

Table 17 shows the average accuracy score and the ROC AUC score. Table 18 illustrates the F1-score for each class and utilized methods. The reader can observe the superior performances of the transformers against the baseline approaches. Furthermore, for the transformer approach, we depict in Fig. 9 the confusion matrix including the summed values across all the folds.

**Table 17:** Classification results in terms of accuracy and ROC AUC score on the classical dataset averaged over all the classes.

	**Accuracy score**	**ROC AUC score**
Nearest Neighbour	0.58	0.61
Random Forest	0.73	0.81
Support Vector Machine	0.50	0.56
LSTM	0.81	0.82
CNN	0.84	0.86
Transformer-based	0.86	0.88

**Table 18:** Classification results in terms of F1-score on the classical dataset.

	**Albanez**	**Beethoven**	**Mozart**	**Macro Average**	**Weighted Average**
Nearest Neighbour	0.18	0.66	0.51	0.45	0.53
Random Forest	0.83	0.83	0.66	0.77	0.77
Support Vector Machine	0.30	0.61	0.42	0.44	0.50
LSTM	0.73	0.82	0.85	0.82	0.84
CNN	0.70	0.81	0.82	0.78	0.80
Transformer-based	0.78	0.88	0.87	0.84	0.86

#### Coarse-grained classification

To analyse the performances of our model at coarse-grained level, we took a sample of tracks from each of the three datasets previously mentioned and performed the classification using k-cross validation with k set to 5. The data is represented by different arrays of a fixed length of 512 integer tokens for a total of 5,593 samples. Considering the classes of the original track, the resulting label distribution of these samples is shown in Table 19.

**Table 19:** Classes distribution of the samples for the coarse-grained classification.

**NES**	**Rock**	**Classical**
732	2,252	2,609

At each iteration of the k-fold cross-validation, we have 4,026 samples for the training set, 448 for the validation set, and 1,119 for the test set.

**Figure 10:**
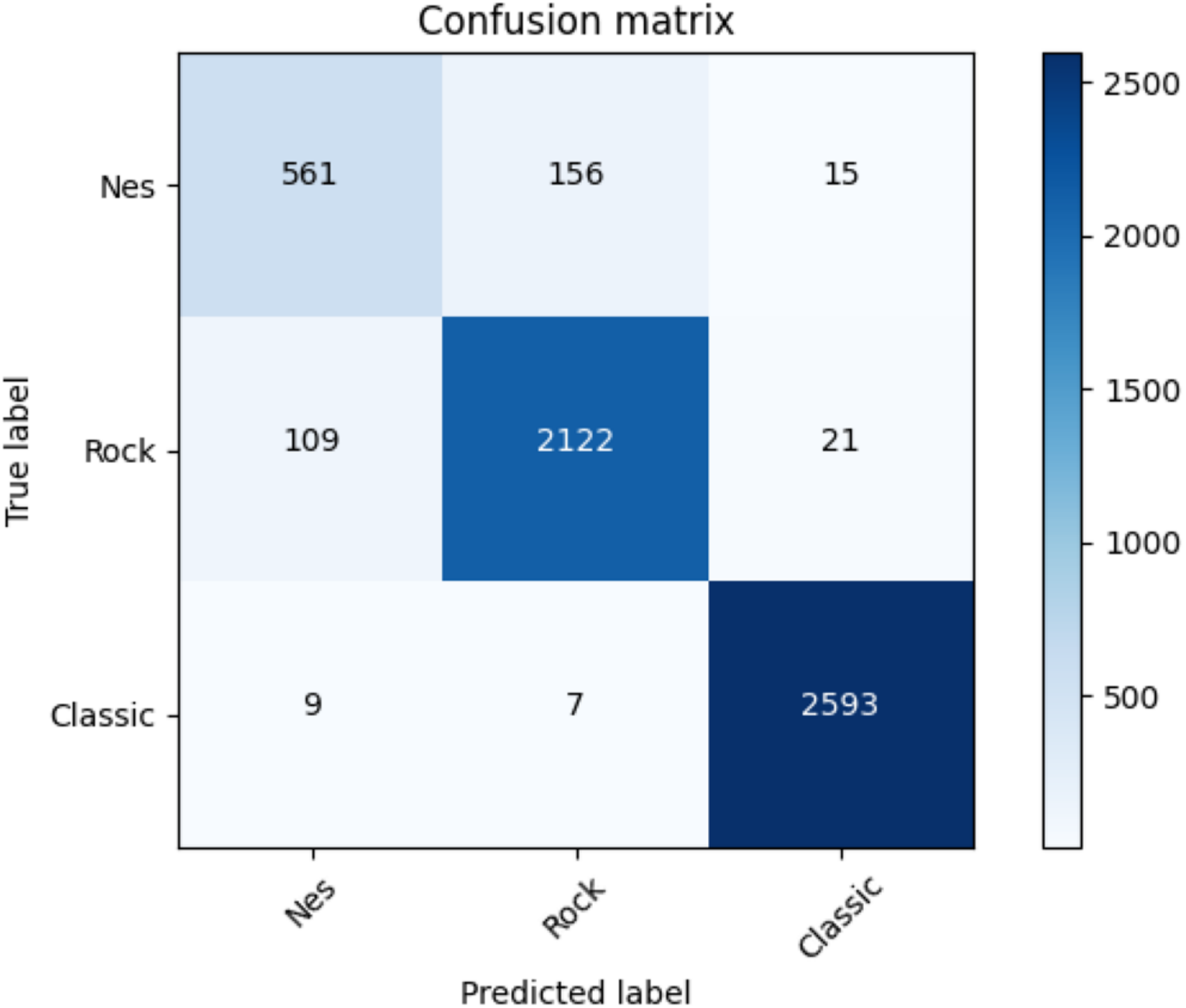
Confusion matrix summed across all the folds for the coarse-grained level classification using the adopted transformers model.

Table 20 illustrates the average accuracy score and the ROC AUC score. Furthermore, Table 21 shows the F1-score for each dataset and adopted method. Even for the coarse-grained level case, the reader can observe the superior performances of the transformers against the baselines. Finally, for the transformer approach, we indicate in Fig. 10 the confusion matrix including the summed values across all the folds.

**Table 20:** Classification results in terms of accuracy and ROC AUC score on the inter-dataset task averaged over the three classes (the three datasets in this case).

	**Accuracy score**	**ROC AUC score**
Nearest Neighbour	0.78	0.75
Random Forest	0.81	0.79
Support Vector Machine	0.81	0.79
LSTM	0.92	0.91
CNN	0.89	0.86
Transformer-based	0.94	0.93

**Table 21:** Classification results in terms of F1-score at coarse-grained level.

	**NES**	**Rock**	**Classical**	**Macro Average**	**Weighted Average**
Nearest Neighbour	0.14	0.77	0.93	0.61	0.76
Random Forest	0.26	0.78	0.98	0.67	0.80
Support Vector Machine	0.22	0.82	0.97	0.67	0.81
LSTM	0.82	0.92	0.97	0.89	0.92
CNN	0.79	0.86	0.90	0.87	0.85
Transformer-based	0.84	0.94	0.99	0.92	0.95

### Results on generation

We adopted a similar language model proposed in Donahue et al. (2019) and fine-tuned it on each different datasets and therefore obtained three different models. We used a Tesla P6 GPU for the fine-tuning process. The new sequences are generated starting from a small excerpt from the intro of a random track present in one of our datasets. This excerpt is called *seed*. The model will complete the *seed* based on the present instruments and notes. Given the relatively small dimension of each dataset used for the fine-tuning, we expect that the final generated track will be a sort of revisitation of the original one where the *seed* comes from. We generated 15 different songs,^5^ 5The generated songs can be freely downloaded from http://192.167.149.18/data.tar.bz2.five from each generative model, and asked five music composers to verify their catchiness with respect to the class of the seed they were generated from. We used a scale of three values (Low, Medium, High) to represent the catchiness. We then mapped the scale to the values (1, 2, 3) and computed the average. Table 22 shows such average results of the five experts on the 15 generated tracks. The Seed column refers to the dataset of the song containing the seed. As it is evident from the table, the catchiness level of all the songs is always close to 3 meaning that in the worst-case scenario (just the track with ID 6) three annotators rated the catchiness with Medium and two annotators rated it with High.

**Table 22:** Catchiness average value of the five experts on the 15 generated tracks.

**Generated Track ID**	**Seed**	**Average Catchiness Level**
1	Rock	2.8
2	Rock	3
3	Rock	3
4	Rock	2.8
5	Rock	3
6	Classic	2.4
7	Classic	3
8	Classic	2.8
9	Classic	2.6
10	Classic	3
11	NES	3
12	NES	3
13	NES	3
14	NES	3
15	NES	3

Moreover, we evaluate our generation process by comparing the sequence created by our model with the original sequence representing the song containing the *seed*. For such a purpose, we employed the local alignment score. The value for such a metric lies within the [0,1] range: 1 when the similarity of the two songs is very high and 0 when it is very low. The reader notices that the seed was not included in the computation. The related results are depicted in Table 23 and indicate that each generated song is different enough from the original seed. This implies that the generated songs consist of patterns not present within the training set.

**Table 23:** Local alignment values on the 15 generated songs.

**Generated Track ID**	**Local Alignment value**
1	0.17
2	0.2
3	0.1
4	0.1
5	0.12
6	0.24
7	0.1
8	0.20
9	0.1
10	0.1
11	0.12
12	0.10
13	0.25
14	0.8
15	0.6

Then, we employed the transformer-based models used for the fine and coarse-grained classification to classify the 15 songs. The goal was to verify whether the generated songs belong to the same class of the track where the seed was selected from (fine-grained) or at least to the same dataset (coarse-grained). Table 24 shows the F1-score for the fine-grained classification. The reader notices that the fine-grained classification performs well for each of the three datasets. The F1-score for NES is the lowest because NES is the dataset with the highest number of classes to guess. Then the rock dataset is next and finally the classical. The F1-score for the coarse-grained classification is 0.95 showing a very high consistency of the generated songs with respect to the genre of their seeds.

**Table 24:** F1-score for fine-grained classification of the 15 generated songs (five for NES, five for rock, and five for classic).

**Dataset used to generate the song**	**F1-score**
NES	0.4
Rock	0.6
Classical	0.8

## Conclusions

In this article, we have performed classification and generation tasks on MIDI music. First, we have described the MIDI file and how it is structured. Then we described the datasets we collected: NES soundtracks, rock music, and classical pieces. In each of them, we tested a classification and generation task. The classification has been performed at fine and coarse-grained levels. At fine-grained level meaning that the classifier had to identify the labels of the MIDI samples for each dataset (*i.e.,* categories of games for NES, which band the underlying rock sample belongs to, and which composer the underlying classical sample belongs to). Next, we merged together the three datasets and addressed a classification task at coarse-grained level. In such a scenario, the classifier had to predict one of the three classes for each test sample (NES, rock, classic). To actually perform the classification, we made use of a transformers-based neural network that has been compared against different classical machine learning and deep learning baselines. In each case of classification, the transformers-based approach outperformed the competitors. Finally, we also tackled the generation task in each dataset. The produced samples were tested using the catchiness values given by five annotators with musical expertise, the local alignment score which indicated whether the generated samples were realistic, and the classification at fine and coarse-grained levels to check whether the generated songs belong to the same class of the songs where the seed was selected from. As future directions, we would like to increase our collection of MIDI files and let the transformer train on a much larger size of samples for both classification and generation. Moreover, to improve the classification, we will employ an ensemble of different transformer models and use majority voting to decide the winning class. One more analysis will be to study the different tracks by instruments and check whether tracks containing a fewer number of instruments (or a particular instrument) are easier to classify or to be used to generate new MIDI samples.

## Additional Information and Declarations

### Data Availability

The following information was supplied regarding data availability: The data and source code are available at Zenodo: aibba19, & simone-angioni. (2023). simone-angioni/midi: Midi Classification & Generation V1.2 (V1.2). Zenodo. https://doi.org/10.5281/zenodo.7786756.
